# Obituary: Remembering Michael Piatak, Jr.

**DOI:** 10.1186/s12977-014-0092-x

**Published:** 2014-12-16

**Authors:** Jeffrey D Lifson

**Affiliations:** AIDS and Cancer Virus Program, Leidos Biomedical Research, Inc., Frederick National Laboratory, Frederick, MD 27102 USA

**Figure 1:**
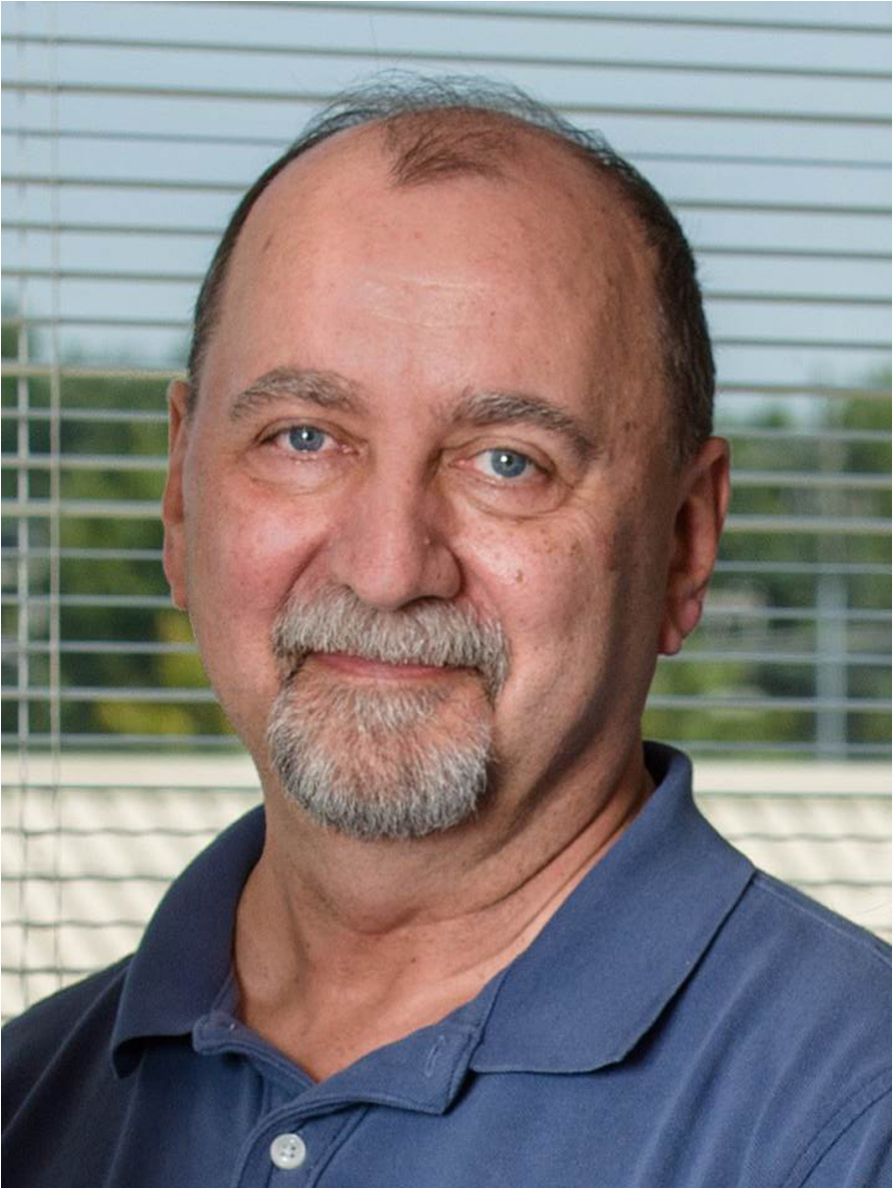
**Michael Piatak, Jr., 2014.**

Michael Piatak, Jr, a talented, creative molecular virologist best known for his many contributions to developing methods for quantitative analysis of retroviral nucleic acids, analyses central to many key advances in AIDS research, passed away suddenly on 19 September 2014 at the age of 64 (Figure 1). His loss will be deeply felt by the research community that benefitted profoundly from his work over the past three decades, and from their rewarding personal interactions with him over his career.

A proud undergraduate alumnus of Penn State (and lifelong Nittany Lion football fan), Mike did his graduate studies at Yale, receiving his PhD in Molecular Virology in 1978, then continuing postdoctoral work at Yale with Sherman Weisman, characterizing SV-40 transcripts and replication in infected cells. Leaving Yale in 1982, he moved to the Bay Area to join the early generation biotechnology company Cetus Corporation. There, in support of programs to generate monoclonal antibody-based therapeutic immunotoxins, he cloned, characterized, and evaluated wild type and mutant forms of a number of different plant derived ribosome inactivating proteins, including pokeweed antiviral proteins, ricin, and abrin, also developing recombinant systems for their expression. It is a sign of the changed times in which we live that the federal agency most likely to be interested in such activities today would be the Department of Homeland Security and not the Food and Drug Administration! Based on his expertise with the cloning and expression of plant derived ribosome inactivating proteins, Mike was recruited in 1987 to join a small Bay Area biotech startup, Genelabs Incorporated, which is where I first met him, beginning a close professional and personal relationship that would extend over the next three decades. At a time when therapeutic options for treating HIV infection were limited, and unconventional therapeutic approaches were being pursued, the company was investigating the potential utility of a plant derived protein, trichosanthin, which had a long history of use in Chinese traditional medicine and showed intriguing antiviral activity in some in vitro HIV assay systems. Although trichosanthin ultimately proved not useful for treating HIV infection, becoming a footnote in the history of development of AIDS drugs, its evaluation triggered developments that have had a lasting impact on the field.

As a small underfunded startup, the company had neither the time nor money to support a large clinical trial that could yield clinical endpoints to demonstrate drug activity. Coming up with a means to assess drug activity other than clinical endpoints was imperative. However, at the time, surrogate markers for in vivo drug activity or efficacy remained controversial, and available virologic readouts such as viral culture, or plasma HIV p24 antigen levels were low to unmeasurable in many HIV infected patients. It seemed like recently developed polymerase chain reaction (PCR) methods could provide the sensitivity necessary to detect the virus, but at the time, most considered PCR to be a qualitative method only, with the exponential amplification inherent to the technique also amplifying the impact of small experimental vagaries to the point that the phrase “quantitative PCR” was viewed as oxymoronic. With standard PCR methods, there was not a consistent, predictable relationship between the amount of amplified product at the end of the reaction and the starting input amount of target template prior to amplification. Nevertheless, demonstrating the characteristics of open mindedness, incisive critical and analytical thinking, and dogged persistence that characterized the rest of his scientific work, Mike set out to see if he could come up with a way to tame PCR in a manner that would allow it to be used for quantifying HIV RNA and DNA.

Impressed by a publication from Gary Gilliland and Frank Bunn, in which they used a matched but internally mutated and readily discriminated target template as an internal control in a “competitive PCR” approach to try to quantify levels of cytokine transcripts, basing quantitation on the relative amounts of the two products in a titration approach rather than absolute quantitation, Mike worked to adapt this concept to quantifying HIV targets, a task that entailed the additional challenge of accommodating the sequence divergence in clinical isolates of HIV. After much development effort and validation, we were satisfied that we had a method, which we designated “quantitative competitive PCR” (QC-PCR), that with control samples could sensitively and accurately quantify HIV nucleic acids, publishing the method in BioTechniques in 1993. The key challenge was whether this method could reliably be applied to clinical samples. We established a collaboration with George Shaw and Mike Saag, who had a large collection of impeccably pedigreed clinical samples and conducted a blinded analysis. As detailed in a benchmark publication in Science in 1993, the assay worked, detecting and quantifying viral RNA in the plasma of all patients tested, including asymptomatic patients in whom HIV p24 antigen and viral culture assays were negative. Our determined viral RNA levels correlated with disease stage and CD4+ T cell counts, and showed readily demonstrable decreases in longitudinal specimens over the course of resolution of primary infection or short term AZT treatment. At a time when much of the field equated the asymptomatic clinically latent phase of HIV infection with virologic latency, based on negative results in HIV p24 antigen and viral culture assays, this publication, arguably more than any other, shifted the conceptual paradigm of HIV pathogenesis, recasting it as a continuous battle of attrition between viral replication and the immune system, and setting the stage for the role of nucleic acid based virologic assessment of new therapies as new drugs and combination therapies became available.

Following on the HIV QC-PCR publications, in response to a request of whether we could provide a similar assay for quantifying SIV in nonhuman primate (NHP) studies, initially to assist in the analysis of a particular vaccine experiment, Mike developed a SIV QC-PCR assay, which we used to help Vanessa Hirsch and Bernie Moss demonstrate modulation of virologic outcome in a study of a Modified Vaccinia Ankara based vaccine, modulation which correlated with prolonged survival, despite a failure to achieve sterilizing protection. This set the stage for decades of subsequent key contributions to AIDS research in NHP models, providing quantitative virologic analyses for numerous investigators across the country with increasingly more sensitive and sophisticated assays, including early adoption of higher throughput real time quantitative PCR methods when the necessary reagents and instrumentation became available, and the more recent development of exquisitely sensitive methods to meet the emerging demands of evaluating HIV cure strategies in NHP models.

Mike was a rare scientist, more interested in coming up with a better way to solve an analytical challenge with a new assay once someone had convinced him that such an assay was important for addressing a significant issue in AIDS research, than with trying to decide himself what the interesting questions were that needed to be addressed. And he was far more interested in answering such questions than worrying about who got credit for answering them. His pragmatism for getting the job done however, was tempered by a strong instinct, whenever possible, to prefer the more elegant solution to a given problem. The only times we ever disagreed professionally were when I felt that a newly developed assay, version n, was ready to be applied to real samples from the actual experiment that had driven the development of the assay, and Mike felt yet another refinement, version n + 1, could make it even better. He was almost always right.

Both Mike and I left California for Maryland in 1995, with Mike doing a 2 year stint working on DNA Diagnostics at Becton Dickinson before rejoining me where I had relocated to the AIDS and Cancer Virus Program (then the AIDS Vaccine Program) at the Frederick National Laboratory (then known as the National Cancer Institute at Frederick). In keeping with the mission of the Frederick National Laboratory to serve as a national resource for the research community, for the next 17 years Mike provided investigators with unparalleled quantitative virologic support of studies in NHP models, and more recently in various clinical studies that demanded ultrasensitive analyses of specimens from HIV infected patients. Beyond the technical rigor, innovative creativity and deep analytical insight that Mike brought to the development and application of assays to support these studies, often developing custom analyses to address study specific requirements, his efforts were characterized by an extraordinary commitment to meeting the needs of collaborating investigators, whether for development of new, seemingly impossible assays to meet emerging experimental requirements, or delivering data to meet unreasonable short notice deadlines tied to submission of grants, abstracts, or manuscripts. Indeed, with reference to his commitment to supporting increasingly demanding needs of collaborators, it was half in jest and half as a cautionary tale that I gave Mike a copy of the classic children’s book If You Give a Mouse a Cookie, which begins “If you give a mouse a cookie, he's going to ask for a glass of milk. When you give him the milk, he'll probably ask you for a straw. When he's finished, he'll ask you for a napkin”. then continues on in kind for many more pages! The book wound up being prominently displayed in Mike’s lab, but the collaborators always got their cookie, and their milk, with a straw, and usually with a nicer napkin than they were expecting, because Mike understood the importance of answering the questions they were posing. Perhaps nowhere was this as true as in his painstaking efforts in recent collaborative studies with Louis Picker for which he developed exquisitely sensitive assays to detect any residual virus in tissues from macaques in which we were able to show that responses to a CMV-vectored SIV vaccine were capable of controlling and ultimately clearing an established infection with a pathogenic SIV. Leading by example, he inspired the same level of dedication and commitment in the members of his laboratory.

Mike’s generosity extended to training investigators from other laboratories in quantitative PCR methods, sharing reagents, expertise and troubleshooting advice. Indeed the one bittersweet silver lining in having the sad task of notifying colleagues across the country of Mike’s sudden passing was the flood of e-mails I received in return, virtually all variations on the tripartite theme of sad, shocked disbelief, grateful acknowledgement of the many key enabling contributions Mike had made to their work, and comments that in addition to their appreciation of his professional contributions, it was Mike’s personal characteristics of warm, good natured, patient, generous, thoughtful humanity that they will remember and miss.

Mike brought those same attributes to his deep involvement in his community and multiple charitable organizations, and his commitment to his family. Indeed, just days before Mike died, his daughter, enrolled in a master’s degree program, was scheduled to give her first ever conference presentation at a meeting in Florida. She was nervous, in part because it was her first presentation and she was the most junior and inexperienced presenter on the program. Mike supported and coached her from a distance, and much to her delight, moments after concluding her successful presentation, her cell phone rang with a surprise congratulatory phone call from her dad, who, in the midst of an incredibly hectic, demanding schedule, had found the time to look up the conference presentation schedule online and make sure to call her to celebrate just after she finished her presentation. That was the kind of man he was, in the lab and at home, and he will be profoundly missed.

